# Functional Screening of Parkinson’s Disease Susceptibility Genes to Identify Novel Modulators of α-Synuclein Neurotoxicity in *Caenorhabditis elegans*

**DOI:** 10.3389/fnagi.2022.806000

**Published:** 2022-04-27

**Authors:** Roman Vozdek, Peter P. Pramstaller, Andrew A. Hicks

**Affiliations:** Institute for Biomedicine, Eurac Research, Affiliated Institute of the University of Lübeck, Bolzano, Italy

**Keywords:** Parkinson’s disease, α-synuclein, GWAS, neurodegeneration, genetic screen, *C. elegans*

## Abstract

Idiopathic Parkinson’s disease (PD) is characterized by progressive loss of dopaminergic (DA) neurons during aging. The pathological hallmark of PD is the Lewy body detected in postmortem brain tissue, which is mainly composed of aggregated α-Synuclein (αSyn). However, it is estimated that 90% of PD cases have unknown pathogenetic triggers. Here, we generated a new transgenic *Caenorhabditis elegans* PD model *eraIs1* expressing green fluorescent protein- (GFP-) based reporter of human αSyn in DA neurons, and exhibited a nice readout of the developed αSyn inclusions in DA neurons, leading to their degeneration during aging. Using these animals in a preliminary reverse genetic screening of >100-PD genome-wide association study- (GWAS-) based susceptibility genes, we identified 28 orthologs of *C. elegans* and their inactivation altered the phenotype of *eraIs1*; 10 knockdowns exhibited reduced penetrance of αSyn:Venus inclusions formed in the axons of cephalic (CEP) DA neurons, 18 knockdowns exhibited increased penetrance of disrupted CEP dendrite integrity among which nine knockdowns also exhibited disrupted neuronal morphology independent of the expressed αSyn reporter. Loss-of-function alleles of the five identified genes, such as *sac-2*, *rig-6* or *lfe-2*, *unc-43*, and *nsf-1*, modulated the corresponding *eraIs1* phenotype, respectively, and supported the RNA interference (RNAi) data. The Western blot analysis showed that the levels of insoluble αSyn:Venus were not correlated with the observed phenotypes in these mutants. However, RNAi of 12 identified modulators reduced the formation of pro-aggregating polyglutamine Q40:YFP foci in muscle cells, suggesting the possible role of these genes in cellular proteotoxicity. Therefore, modulators identified by their associated biological pathways, such as calcium signaling or vesicular trafficking, represent new potential therapeutic targets for neurodegenerative proteopathies and other diseases associated with aging.

## Introduction

Neurotoxicity of disordered α-synuclein (αSyn) is a pathogenetic hallmark of synucleinopathies, including Parkinson’s disease [PD; Spillantini et al. (1997)]. There are more than 20 reported genes associated with monogenic parkinsonism, including autosomal dominant *SNCA* encoding alpha-synuclein (αSyn) and *LRRK2* encoding Leucine-rich repeat kinase 2, or autosomal recessive *PRKN* encoding the E3 ubiquitin ligase Parkin and *PINK1* encoding Phosphatase and tensin homologue (PTEN)-induced kinase (Blauwendraat et al., 2020). However, familial PD with the identified genetic variants still accounts for only about 10% of diagnosed PD cases, while pathogenetic triggers in sporadic forms of PD are largely unknown (Reeve et al., 2014). Therefore, it is largely considered to be a complex disease with multifactorial etiology. In recent years, several genome-wide association studies (GWASs) have identified many tens of risk signals associated with sporadic PD surrounded by hundreds of potential susceptibility genes (Nalls et al., 2019). To date, there is little to no published functional validation of genes in these loci. In this study, we examined the role of approximately 100 of these genes in maintaining dopaminergic (DA) neurons upon exogenous expression of human αSyn in a newly constructed *Caenorhabditis elegans* genetic model designed to aid rapid initial functional screening.

The roundworm *C. elegans*, which does not possess the gene for αSyn, has been well-established as a PD model, which can help identify genes that protect against exogenous αSyn-induced degeneration of DA neurons or aggregation of αSyn in muscle cells (Cooper and Van Raamsdonk, 2018; Koopman et al., 2019). The *C. elegans* PD model, expressing αSyn tagged with green fluorescent protein (GFP) in the body wall muscle cells, was used to seek the effectors of αSyn misfolding through reverse genetic screens elicited by RNA interference (RNAi) (Hamamichi et al., 2008; van Ham et al., 2008; Jadiya et al., 2016). In addition, RNAi of 1,673 genes related to neuronal function revealed genes of the endocytic pathway in pan-neuronal αSyn-induced growth/motor abnormalities (Kuwahara et al., 2008). αSyn expressed in nematode DA neurons causes DA neurodegeneration characterized by neuronal loss or abnormal dendritic processing, and also dopamine-mediated locomotion deficits (Lakso et al., 2003; Kuwahara et al., 2006). In addition, in these PD worm models, the expression of human Torsin A and yeast Rab1, which play a role in vesicular trafficking, showed neuroprotective activity against α-synuclein-induced degeneration (Cao et al., 2005; Cooper et al., 2006). Several studies have also demonstrated that fluorescence-based reporters of αSyn expressed in neuronal tissues recapitulate DA neuronal deficits and show the spread of αSyn into the epithelium (Cooper et al., 2018; Sandhof et al., 2020). However, none of these previous nematode models have been used to identify modulators of αSyn aggregation in neurons. Here, we have generated a new nematode model, *erals1*, which allows monitoring of the expression of αSyn in DA neurons *in vivo*, and used these transgenic animals for functional screening of identified PD risk genes.

## Materials and Methods

### *Caenorhabditis elegans* Strains

Unless otherwise stated, animals were maintained by standard procedures on nematode growth media (NGM) plates. Transgenic strains were generated by germline transformation using microinjection into Bristol strain N2. *C. elegans* constructs for the *eraIs* transgene were generated by direct PCR of the human *SNCA* gene cloned into the pDEST vector in front of, and in frame with, the Venus reporter gene with *unc-54* 3′UTR. The pDEST vectors carrying hSNCA:Venus or mCherry were subsequently recombined with a pENTRY vector carrying the *dat-1* promoter sequence. Transgenic constructs were co-injected at 50 ng/μl, and stable extrachromosomal lines of mCherry- and Venus-positive animals were established. The extrachromosomal array was subsequently integrated by ultraviolet (UV) irradiation, and the lines carrying *eraIs1* were subjected to 5× outcrossing. The strains used were as follows: *eraIs1*, *otIs181*, *eraIs1;otIs181, eraIs1;pdr-1(gk448), eraIs1;pink-1(tm1779), eraIs1;wlzIs3*, *eraIs1;lfe-2(sy326)*, *eraIs1;unc-43(n1186)*, *eraIs1;nsf-1(ty10)*, *eraIs1;sac-2(ok2743)*, *eraIs1;unc-32(e189)*, *eraIs1;rig-6(ok1589)*, and *rmIs133 (unc-54p:Q40:YFP)*.

### Reverse Genetic Screen

RNAi was fed to worms to knockdown the respective gene function. Gravid animals carrying *eraIs1*, *otIs181*, or *rmsIs133* transgenes were placed on NGM media containing ampicillin 25 μg/ml and 1 mM IPTG and seeded with bacteria producing the desired double-stranded RNA (dsRNA). Progenies were subsequently grown at 23°C till the fourth larval stage (L4) stage in which the phenotype was scored *via* visual examination. *eraIs1* animals were scored for the disruption of cephalic (CEP) integrity, which was defined by the presence of fluorescent inclusions in the area of the CEP axons (phenotype A) and CEP dendrite blebbing/loss (phenotype B). Both of these phenotypes were selected as being the most tractable by visual inspection due to the bright fluorescence of the αSyn reporter in CEP neurons, which allowed quantification of individual RNAi knockdowns in a relatively high-throughput way. Moreover, assessing animals at the L4 stage revealed modulators of phenotypes A and B. Each population of L4 knockdowns having 0–60% of individuals exhibiting phenotype A and 40–100% of individuals exhibiting phenotype B was classified as modulators. *otIs181* and *rmsIs133* animals were scored for the disruption of CEP integrity, defined by dendrite blebbing/loss and number of fluorescent foci, respectively. At least 20 *eraIs1*, 20 *otIs181*, or 3 *rmsIs133* animals were visually examined for penetrance and fluorescent foci quantification, respectively. Visual examination was done using a fluorescent stereoscope (Nikon SMZ800N) by one researcher with coded plates to ensure blindness of the investigator. The results were recorded and subsequently decoded to reveal the names of RNAi targets. Bacterial clones were obtained from the *C. elegans* RNAi collection—Ahringer (Source: Bioscience).

### Locomotor Assay

Animals were grown at 23°C under non-starved conditions. One-day-old adult hermaphrodites were placed on NGM plates and recorded. For the crawling/swimming transition assay, animals were subjected to liquid exposure by dropping 30 μl of M9 buffer on the plate, which was dried within 7–8 min. For the mechanical stimuli assay, animals crawling on the NGM plate with seeded bacteria were stressed by five taps of the plate on the bench. For the foraging assay, well-fed animals were placed 1 cm away from the bacterial lawn. Animal movement was subsequently screened by quantifying body bends in the indicated time intervals of 30 s. During crawling, body bends were scored as head turns for moving forward. During swimming, body bends were scored as C-shaped movements. At least three biological replicates (five animals per assay) were used for statistical analysis. To compare the distribution of animals on and off the food area, 1-day old adults, which were starved for 1 h, were placed 1 cm away from the bacterial lawn on NGM plates, and their position on the plate was scored 30 min later. At least 50 animals for each group (N2 vs. *eraIs1*) with three independent biological replicates were used for statistical analysis.

### Statistical Analyses

Data are presented as mean ± standard deviation (SD) with *p*-values calculated by one-way analysis of variance (ANOVA) with Bonferroni correction for multiple comparisons and the Mann–Whitney test for single comparison.

### Determination of α-Synuclein Levels

Worms were grown at 23°C as described above and collected as a mixed population of all larval and adult stages. Wet worm pellets were subsequently frozen at −20°C for 4 h to disrupt the nematode cuticle. Worm lysates were prepared by sonication of worm pellets resuspended in M9 buffer containing protease and phosphatase inhibitor cocktail. Crude extracts were immediately centrifuged for 1 h at 4°C and 20,000 *g*, and the supernatants (soluble fraction) and pellets (insoluble fraction) were used for the determination of αSyn:Venus levels in the indicated mutants by the Western blot analysis. Samples were boiled for 10 min in reducing lithium dodecyl sulfate (LDS) sample buffer and submitted to SDS-PAGE (4–12% precast gradient gel). Protein immunodetection was performed by the Western blot using a custom-made mouse monoclonal anti-αSyn antibody (Abnova, MAB5383 1:2,000). Ubiquitin, which was detected using a mouse monoclonal anti-ubiquitin Ab (CellSignal, P4D1 1:2,000), along with an unspecific signal, was used to demonstrate protein loading. Western blot signals were semi-quantified in ImageJ software (Fiji).

### Imaging

For confocal imaging, animals were mounted on a 2% agarose pad with 10 mM sodium azide and imaged on a Leica SP8-X confocal laser scanning microscope within 2–10 min. At least three images representing each *C. elegans* strain from three independent biological replicates were analyzed.

## Results

**FIGURE 1:**
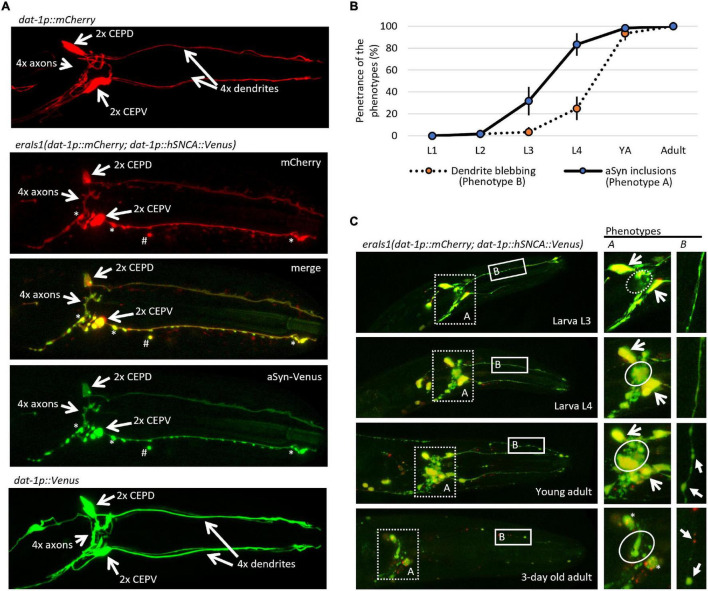
*eraIs1* animals exhibit progressive formation of α-Synuclein (αSyn) inclusions and disruption of the dopaminergic (DA) neuron integrity during aging. **(A)** Representative fluorescent image of the head of third larval stage (L3) animals expressing the mCherry reporter alone, the mCherry reporter with the αSyn:Venus reporter, and the Venus reporter alone in DA neurons. Two head ventral CEPV and two dorsal CEPD neurons and their axons are indicated by open arrows. Stars indicate αSyn:Venus inclusions. Hashtag indicates extruded αSyn:Venus from DA neurons. **(B)** Distribution of phenotypes quantified at distinct developmental stages of *eraIs1* animals. *n* ≥ 20 total animals for each group with three independent biological replicates. Mean ± standard deviation (SD) is shown. **(C)** Representative fluorescent images of the head of *eraIs1* animals at different stages of development. Dotted square line highlights the area used for the quantification of phenotype A (αSyn:Venus inclusions) and the solid square line highlights the area used for the quantification of phenotype B [cephalic (CEP) dendrite blebbing]. On the right, there are enlarged images of the phenotypes A and B areas that show the phenotype identifiers for quantification. The circle line defines the area for the evaluation of phenotype A. The dashed circle indicates a normal phenotype, and the solid circle indicates phenotype A. Open arrows indicate CEP cell bodies. Three-day-old adult exhibits rounded CEP cell bodies indicated by the stars. Phenotype B is indicated by arrows.

To study neuronal genes that mediate proteostasis upon the formation of αSyn inclusions, we have generated a new transgenic *C. elegans* carrying *eraIs1(dat-1p:human SNCA:Venus; dat-1p:mCherry)*. The *eraIs1* transgene uses the *dat-1* promoter to drive the expression of a human *hSNCA:Venus* reporter along with an mCherry reporter specifically in DA neurons consisting of four CEP neurons, two anterior deirids (ADE) in the head, and two posterior deirids (PDE) in the tail. Bright GFP-based reporter Venus of human αSyn allowed us to monitor the spatial and temporal formation of its inclusions (fluorescent foci) in axons, dendrites, and cell bodies (Figure 1A). Notably, we observed that expressed mCherry, which is diffusely distributed in the cytosol under standard conditions throughout development and aging, forms inclusions in the presence of αSyn:Venus. Similar to the mCherry reporter, the expression of the untagged Venus reporter alone did not form fluorescent inclusions, indicating that the expression of exogenous human αSyn is crucial for the formation of inclusions (Figure 1A). Intriguingly, both αSyn:Venus and mCherry reporters in *eraIs1* can also exhibit aggregated fluorescent signals away from intact neurons (Figure 1A). These isolated fluorescent foci of aggregated proteins presumably represent an extruded toxic material as an active neuronal self-maintaining mechanism against disrupted proteostasis rather than cell remnants of degenerated neurons (Melentijevic et al., 2017).

During visual inspection of *eraIs1* animals using a fluorescent stereoscope, we observed that *eraIs1* nematodes exhibit the progressive formation of αSyn:Venus inclusions accompanied by morphological changes of CEP neurons during development, and ultimately the disruption of neuronal integrity in aged animals. The bright Venus fluorescence allowed us to determine which animals developed fluorescent inclusions in the axons of CEP neurons (phenotype A) and pathological morphology (blebbing) of CEP dendrites (phenotype B). We assessed the penetrance of these two phenotypes during animal development and determined that the penetrance of phenotype A increases from 32% at the third larval stage (L3) to 83% at L4, while the penetrance of phenotype B reaches 25% at L4 and 93% at the young adult stage. In 3-day-old adults, CEP neurons exhibited rounded cell bodies accompanied by dendritic disorganization, indicating the process of neurodegeneration associated with aging (Figures 1B,C; Chen et al., 2015).

**FIGURE 2:**
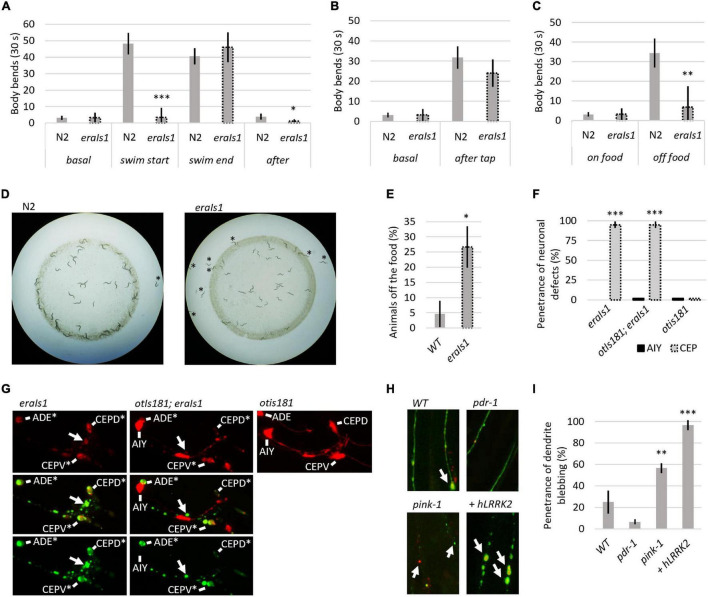
Evaluation of the eraIs1 model. **(A)** Locomotor phenotype of the indicated animals placed on a nematode growth media (NGM) plate seeded with OP 50 bacteria (basal), right after exposure to M9 buffer (swim start), 5 min after exposure to M9 (swim end), and right after swimming (after). *n* ≥ 10 young adults for each group with three independent biological replicates. During crawling, body bends were scored as head turns for moving forward. During swimming, body bends were scored as C-shaped movements. Mean ± SD is shown. *p < 0.05. ^***^p < 0.001. **(B)** Locomotory phenotype of the indicated animals localized in the bacterial lawn (basal) and right after being disturbed by tapping (after tap). *n* ≥ 10 young adults for each group with three independent biological replicates. Mean ± SD is shown. **(C)** Locomotory phenotype of indicated animals localized in bacterial lawn (on-food) and right after being placed off the bacteria (off-food). *n* ≥ 10 young adults for each group with three independent biological replicates. Mean ± SD is shown. ^**^p < 0.01. **(D)** Distribution of the indicated animals on the NGM plate seeded with bacteria. Animals starved for 1 h are captured 30 min after being placed 1 cm away from the bacterial lawn. **(E)** Percentage of the indicated animal populations found outside the bacterial lawn. *n* ≥ 50 young adults for each group with three independent biological replicates. Mean ± SD is shown. *p < 0.05. **(F)** Percentage of young adult *eraIs1*, *otIs181;eraIs1*, and *otIs181* animals exhibiting αSyn:Venus and mCherry inclusions in CEP and AIY neurons. *n* ≥ 20 animals for each group with three independent biological replicates. Mean ± SD is shown. ^***^p < 0.001. **(G)** Enlarged fluorescent images of the head of young adult *eraIs1*, *eraIs1;otIs181*, and *otIs181* animals. Arrows indicate αSyn:Venus inclusions. CEP and AIY cell bodies are indicated. Stars indicate neuronal cell bodies with the αSyn:Venus and mCherry inclusions. **(H)** Representative fluorescent images of CEP dendrites in the indicated *eraIs1* animals. *eraIs1* wild type (WT), *eraIs1;wlzIs3* carrying *human LRRK2 p.G2019S*, and *pdr-1(gk448)* and *pink-1(tm1779)* mutants are shown. Arrows indicate CEP dendrite blebbing. **(I)** The penetrance of dendrite blebbing in the indicated *eraIs1* animals. *n* ≥ 20 fourth larval stage (L4) animals for each group with three independent biological replicates. Mean ± SD is shown. ^**^p < 0.01. ^***^p < 0.001.

Dopaminergic neurons regulate several animal behaviors, such as locomotory response to food availability (Sawin et al., 2000; Omura et al., 2012), foraging (Hills et al., 2004), or movement transitions between crawling and swimming (Vidal-Gadea et al., 2011). To determine whether *eraIs1* animals exhibit respective behavioral phenotypes due to DA neuronal defects, we exposed 1-day-old adult *eraIs1* and wild-type isolate N2 to various stimuli and compared their locomotor behavior by counting the body bends used for crawling or swimming. First, we exposed well-fed animals, crawling on the bacterial lawn on NGM plates, to M9 buffer. While N2 animals exposed to M9 liquid buffer responded immediately by swimming at the rate of 48 C-shaped body bends per 30 s, *eraIs1* animals failed to respond, with an average rate of 3 C-shaped body bends per 30 s (Figure 2A). In addition, while crawling locomotion of well-fed *eraIs1* animals was not altered before M9 buffer exposure, it was noticeably reduced after swimming compared to N2 animals (Figure 2A). The defective response of *eraIs1* to M9 buffer is not caused by a locomotion defect as both N2and *eraIs1* exhibited a swimming phenotype at the rate of 41 and 46 body bends per 30 s after 5 min in M9 buffer, respectively (Figure 2A). Moreover, mechanical stimuli elicited by plate tapping did not reveal significant behavioral changes between N2 and *eraIs1* animals, indicating that the locomotion of *eraIs1* animals is not impaired (Figure 2B). Next, we tested whether *eraIs1* animals would exhibit foraging defects. We placed well-fed animals on an NGM plate outside the bacterial lawn and found that *eraIs1* animals exhibit a reduced locomotor response to lack of food (Figure 2C). Moreover, when we used starved animals and examined the plates after 30 min, we observed that a greater number of *eraIs1* animals remained outside, but in proximity to, the bacterial lawn, indicating a possible food sensing deficit (Figure 2D). Specifically, we observed that only 74% of *eraIs1* animals were located inside the bacterial lawn compared to 95% of N2 animals (Figure 2E). Taken together, these behavioral data demonstrate that *eraIs1* animals exhibit behavioral characteristics similar to that induced by dopamine deficiency.

We verified the effect of αSyn on neuronal integrity with an independent neuronal mCherry reporter. We used the *otIs181* transgene that uses *dat-1:mCherry* together with *ttx-3:mCherry*, which drives its expression in DA neurons and amphid interneurons (AIY), respectively, and crossed it with *eraIs1*. As expected, *eraIs1* induced neuronal defects of the DA neurons but not the AIY neurons in young adults, suggesting cell-autonomous neurotoxicity of the expressed αSyn (Figures 2F,G). To further validate the PD model *eraIs1*, we tested whether known neurodegenerative triggers, such as loss-of-function of Parkin and PINK1 and gain of function of human LRRK2, which are associated with familial forms of PD, would induce neuronal defects upon αSyn expression (Yao et al., 2010; Blauwendraat et al., 2020). We used loss-of-function alleles of *C. elegans prd-1/Parkin*, *pink-1/PINK1*, and the transgene *wlzIs3* expressing human *LRRK2 p.G2019S* (Saha et al., 2009) and crossed them with *eraIs1*. We assessed the toxicity of αSyn in these mutants at the L4 developmental stage, which allowed us to identify both suppressors of phenotype A and enhancers of phenotype B. In addition, the size of L4 animals reduces experimental bias resulting from impaired traceability of respective phenotypes in younger larval stages and phenotypic variability in adults. We found that dendrite blebbing was exacerbated in animals carrying *pink-1* and *wlzIs3* but not in *pdr-1* mutants (Figures 2H,I). These data demonstrate that the phenotype modulated in *eraIs1;pink-1 and eraIs1;wlzIs3* animals could be used as a readout to identify new modulators of αSyn-induced neurodegeneration.

**FIGURE 3:**
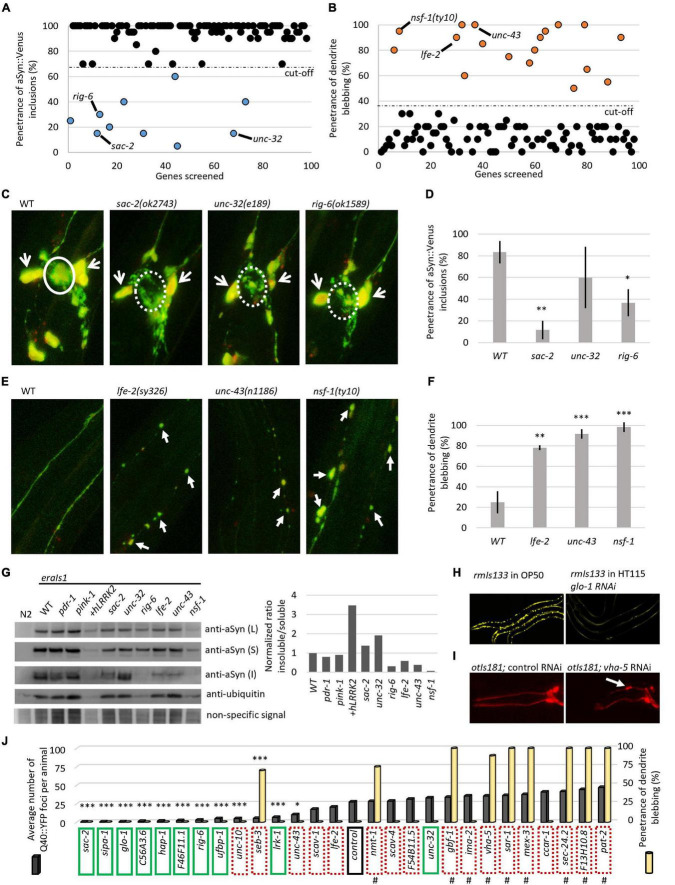
Reverse genetic screening for altered αSyn neurotoxicity in *eraIs1* animals. **(A)** Distribution of the quantified phenotype of αSyn inclusions (phenotype A) in 98 different knockdowns. **(B)** Distribution of quantified phenotype B (CEP dendrite blebbing) in 98 different knockdowns. **(C)** Representative fluorescent images of CEP cell bodies and their axons of *eraIs1* mutants at L4 stage. The solid circle line indicates phenotype A while the dotted circle line indicates a normal phenotype. Open arrows indicate CEP cell bodies. **(D)** Penetrance of phenotype A (αSyn:Venus inclusions) in the indicated mutants. *n* ≥ 20 L4 animals for each group with three independent biological replicates. Mean ± SD is shown. *p < 0.05. ^**^p < 0.01. **(E)** Representative fluorescent images of CEP dendrites of *eraIs1* mutants at the L4 stage. Arrows indicate CEP dendrite blebbing. **(F)** Penetrance of phenotype B (dendrite blebbing) in the indicated mutants. *n* ≥ 20 L4 animals for each group with three independent biological replicates. Mean ± SD is shown. ^**^p < 0.01. ^***^p < 0.001. **(G)** αSyn:Venus protein levels determined by SDS-PAGE followed by western blot using anti-αSyn Ab, and anti-ubiquitin Ab and non-specific signal as a loading control, and the calculated ratio of insoluble/soluble αSyn levels normalized to WT are presented. Animals of mixed stages were collected from three biological replicates and 10 μl of either lysed *Caenorhabditis elegans* pellets of the same density (L) or soluble (S) and insoluble (I) fraction were loaded per lane. *eraIs1* animals carry the following alleles: WT, *pdr-1(gk448)* and *pink-1(tm1779)*, *wlzIs3(snb-1p:hLRRK2 p.G2019S)*, *lfe-2(sy326)*, *unc-43(n1186)*, *nsf-1(ty10)*, *sac-2(ok2743)*, *unc-32(e189)*, and *rig-6(ok1589)*. **(H)** Representative fluorescent images of *rmIs133* animals at the L4 stage expressing Q40:YFP in body wall muscles upon standard (feeding OP50 bacteria) and *glo-1* RNA interference (RNAi). **(I)** Representative fluorescent images of the head of young adult *otIs181* animals upon control and *vha-5* RNAi. **(J)** Quantification of Q40:YFP fluorescent foci in *rmIs133* (*n* ≥ 3 L4 animals) and penetrance of CEP dendrite blebbing in *otIs181* animals (*n* ≥ 20 L4 animals) among RNAi knockdowns. Green boxes indicate identified modulators of phenotype A (αSyn:Venus inclusions in *eraIs1*), dotted red boxes indicate identified modulators of phenotype B (dendrite blebbing in *eraIs1*). Hashtags indicate knockdowns that fail to propagate (lethal or sterile phenotype). Stars indicate genes whose inactivation reduced Q40:YFP foci. *p < 0.05. ***p < 0.001.

We searched several current GWAS databases, such as iPDGC (Grenn et al., 2020), GWAS catalog (Buniello et al., 2019), and PDgene (Lill et al., 2012; Nalls et al., 2014) for genes associated with sporadic PD. Collectively, we identified 98 risk signals at different levels of statistical significance from 89 independent genomic loci. We selected 131 PD susceptibility genes based on their proximity and position relative to the PD risk signals and analyzed them *in silico* for evolutionary conservation in the *C. elegans* genome based on phylogenetic and structural data information. For 97 of the human-associated potential risk genes, we identified at least one *C. elegans* ortholog, collectively consisting of 127 different worm genes (Supplementary Table 1). We set out to perform a preliminary reverse genetic screen of these identified *C. elegans* orthologous genes using an RNA interference by “feeding approach” (genes for which an RNAi clone was not available were not included in the screen), or using previously isolated loss-of-function alleles, for modulated αSyn neurotoxicity in the *eraIs1* strain. Overall, we have visually examined 98 knockdown/mutant animal populations and recorded the penetrance of phenotypes A (αSyn:Venus inclusions in CEP axons) and B (CEP dendrite blebbing/loss) at the L4 developmental stage (Figure 1C). The preliminary RNAi data obtained clustered the examined genes into two basic groups: (i) 10 genes whose inactivation reduced the penetrance of phenotype A (Figure 3A and Table 1) and (ii) 18 genes whose inactivation increased the penetrance of phenotype B (Figure 3B and Table 1).

**TABLE 1:** Identified modulators.

Biological process	Modulators of phenotype A	Presumable function	Modulators of phenotype B	Presumable function
Calcium signaling	*C56A3.6/MICU3*	Regulates mitochondrial Ca2 + uptake (Patron et al., 2019).	*unc-43/CAMK2D*	Regulates Ca2+ homeostatis through targeting T-type calcium channels (Welsby et al., 2003).
			*lfe-2/ITPKB*	Inhibits Ca2+ release into cytosol from endoplasmic reticulum by metabolizing IP3 (Berridge, 2016).
			seb-3/CRHR1***	Inhibits T-type calcium channels through GPCR signaling upon binding corticotropin-releasing factor and urocortin (Bonfiglio et al., 2011).
GTPase activity and vesicle trafficking	*lrk-1/LRRK2*	Phosphorylates Rab family of small GTPases (Steger et al., 2016).	*unc-10/RIMS1*	Regulates exocytosis of synaptic vesicle using Ras GTPase activity (Wang and Südhof, 2003).
	*glo-1/RAB29*	Maintains endosome-trans-Golgi network structure and retrograde trafficking by recruiting LRRK2 (Purlyte et al., 2018).	sec-24.2/SEC24A***	Mediates protein transport from the endoplasmic reticulum by forming coat of the vesicles (Wendeler et al., 2007).
	*unc-32/ATP6V0A1*	Transports protons across cellular membranes to acidify various organelles (Aoto et al., 2021).	vha-5/ATP6V0A1***	Transports protons across cellular membranes to acidify various organelles (Aoto et al., 2021).
	*sipa-1/SIPA1L2*	Orchestrates retrograde trafficking of amphisomes using Rap GTPase activity (Andres-Alonso et al., 2019).	*nsf-1/NSF*	Promotes fusion of the vesicle with the target membrane using ATPase activity (Zhao et al., 2007).
	*sac-2/INPP5F*	Regulates endocytic recycling pathway using PI4P 4-phosphatase activity (Nakatsu et al., 2015).	sar-1/SAR1B***	Initiates coat formation of nascent vesicles using GTPase activity (Hanna et al., 2016).
			nmt-1/NMT2***	Promotes ARF6 GTPase using its lysine myristoyltransferase activity (Kosciuk et al., 2020).
			gbf-1/GBF1***	Maintains Golgi network homeostasis using GEF activity toward ARF GTPases (Kawamoto et al., 2002).
Other	*rig-6/CNTN1*	Regulates neurite outgrowth by mediating cell-cell interactions (Falk et al., 2002).	pat-2/ITGA8***	Regulates neurite outgrowth by mediating cell-cell interactions (Müller et al., 1995).
	*ufbp-1/DDRGK1*	Regulates reticulophagy (Liang et al., 2020).	mex-3/MEX3C***	Promotes mRNA decay (Kuniyoshi et al., 2014).
	*hap-1/ITPA*	Metabolizes ITP and XTP (Simone et al., 2013).	*ccar-1/CCAR2*	Regulates cell cycle and apoptosis (López-Saavedra et al., 2016).
	*F46F11.1/PPIP5K2*	Maintains IP6 and IP7 levels (Fridy et al., 2007).	*Ima-2/KPNA1*	Imports proteins into nucleosome (Moroianu et al., 1995).
			F13H10.8/SPTSSB***	Stimulates the activity of serine palmitoyltransferase (Han et al., 2009).
			*scav-1 and 4/SCARB2*	Serves as lysosomal receptor for protein targeting (Reczek et al., 2007).
			*F54B11.5/RNF141*	-

***Indicates the genes whose inactivation induced dendrite blebbing also in the control strain otIs181 and thus impaired neuronal integrity independently of exogenously expressed α-Synuclein (αSyn).

We validated each RNAi phenotype with six previously characterized loss-of-function alleles, such as *sac-2, rig-6*, and *unc-32*, whose inactivation reduced the penetrance of phenotype A, and *unc-43, lfe-2*, and *nsf-1*, whose inactivation increased the penetrance of phenotype B. We confirmed reduced penetrance of αSyn:Venus inclusions (phenotype A) formed in *sac-2* and *rig-6* mutants even though confocal imaging of these mutants revealed that the formation of inclusions was not completely suppressed. However, the size of the formed inclusions cannot be recognized by visual inspection using a fluorescent stereoscope compared to the fluorescent inclusions accumulated in wild-type (WT) animals, which explains the observed reduction in penetrance in these mutants (Figures 3C,D). Next, we confirmed increased penetrance of dendrite blebbing (phenotype B) in *lfe-2* and *unc-43* that phenocopy *nsf-1* mutants, and confocal imaging revealed the altered integrity of their CEP dendrites (Figures 3E,F). These data show that the preliminary results obtained from reverse genetic screening have been validated for five different genes confirming their modulatory role in αSyn neurotoxicity in *C. elegans*.

We investigated whether the altered penetrance of phenotypes A and B in the tested mutants was associated with altered levels, solubility, or processing of expressed αSyn:Venus. We used Western blot analysis to detect the levels of αSyn:Venus in crude, soluble and insoluble *C. elegans* extracts and the amount of αSyn:Venus its soluble and insoluble fractions. We used a detergent-free lysis buffer in which the insoluble αSyn should be composed of both aggregated/fibrillar form and monomeric αSyn bound to the cellular/organelle membrane. First, we did not detect any significant alterations of normalized αSyn levels in crude extracts of the tested mutants, indicating that modulated phenotypes are not mediated through altered expression of αSyn:Venus but rather to altered capability of neurons to cope with the stress induced by αSyn. However, we found various levels of insoluble αSyn among the tested mutants. We normalized the ratio of insoluble/soluble αSyn to WT and detected higher levels of insoluble αSyn in *unc-32* mutants and in strain co-expressing human LRKK2 p.G2019S with increased penetrance of phenotype B. On the other hand, levels of insoluble αSyn were markedly decreased in *rig-6* mutants, in which penetrance of phenotype A was reduced, and in *nsf-1* mutants, in which the penetrance of phenotype B was increased (Figure 3G). These data suggest that both modulated phenotypes in *eraIs1* mutants might have various pathogenetic basis that did not have to be necessarily associated with aggregation-related nor membrane-bound-related αSyn toxicity.

To better investigate the role of all identified modulators in cellular proteostasis, we next assessed the RNAi of the 28 identified genes for their capability to modulate the aggregation of the pro-aggregating poly-Glutamine (Q40):YFP protein expressed in muscle cells. We used *rmIs133(unc-54p:Q40:YFP)* animals, in which Q40:YFP progressively forms fluorescent foci during development (van Ham et al., 2008) and are easily tractable by visual inspection under a fluorescent stereoscope. We counted the fluorescent foci at the L4 stage among the tested RNAi conditions and found that nine modulators of phenotype A, such as RNAi of *sac-2, sipa-1, glo-1, C56A3.6, hap-1, F46F11.1, rig-6, ufbp-1*, and *lrk-1*, significantly reduced the number of formed Q40:YFP foci (Figures 3H,J). Interestingly, the RNAi against *unc-10*, *seb-3*, and *unc-43*, which were classified as modulators of phenotype B, also reduced the number of Q40:YFP foci (Figure 3J). These data suggest that 12 identified mediators of Q40:YFP aggregation may play a role in the maintenance of proteotoxicity, including neurotoxicity induced by the expression of αSyn in *eraIs1* animals.

To evaluate the possibility that the identified modifiers could modulate neuronal integrity independent of αSyn expression, we retested all identified modulators in animals carrying *otIs181(dat-1p:mCherry;ttx-3p:mCherry)* (Flames and Hobert, 2009) expressing an mCherry reporter in DA neurons alone. We found that RNAi against nine genes that were identified as modulators of phenotype B, including *seb-3, sec-24.2, vha-5, sar-1, nmt-1, gbf-1, pat-2, mex-3*, and *F13H10.8*, induced CEP dendrite blebbing in *otIs181* animals, indicating that inactivation of these genes disrupted neuronal integrity independent of neurotoxic αSyn expression (Figures 3I,J, and Table 1). Notably, RNAi of some of these genes caused sterility, larval arrest, or developmental delay of animals, indicating a widespread effect of gene inactivation on worm health. The complementary screens with *eraIs1* and *otIs181* animals identified 19 genes, whose inactivation modulated αSyn-mediated toxicity, and other nine genes, whose inactivation disrupted the integrity of DA neurons independent of αSyn-mediated toxicity.

## Discussion

Several studies over the years have revealed tens of genes that can modulate αSyn aggregation and associated neurodegeneration in various animal and cell model systems. However, a model that allows monitoring of the αSyn processing in neurons *in vivo* in a high-throughput way under various conditions is, until now, somewhat missing. Here, we generated a *C. elegans* model, in which αSyn inclusions and accompanying neuronal morphological processes can be monitored under a fluorescent stereoscope *in vivo*. We showed that the formation of αSyn inclusions was predominantly detected in the axons of CEP neurons at the L4 larval stage, which allowed the screening of inactivated genes that reduced the level of inclusion formation. We also showed that the expression of αSyn in *eraIs1* induced the dendrite blebbing of CEP neurons in adults but was rare in larval stages, which allowed the screening of the genes, whose inactivation induced dendrite blebbing in the larval stages. Therefore, this study provides the first systematic functional screening of the genes identified in the PD GWAS data and reveals new genetic pathways that could mediate PD pathogenesis.

First, we identified 18 genes, whose inactivation exacerbated αSyn:Venus-induced neuronal defect characterized by CEP dendrite blebbing. Notably, RNAi of nine of these modulators induced dendrite blebbing independent of αSyn:Venus expression in these neurons and/or caused impaired propagation of animals, which indicates their crucial role in neuronal cell integrity and *C. elegans* biology. Therefore, we hypothesize that these genes, when misregulated, might induce neuronal defects in other organisms independent of the pathogenicity of αSyn. These data also raise speculation whether some PD GWAS genes could play a role in PD progression in an αSyn-independent manner. Second, we identified 10 genes whose inactivation by RNAi reduced the formation of αSyn:Venus inclusions in CEP axons.

RNAi screening identified a total of 19 genes, which, when inactivated, modulated αSyn-related toxicity; however, our data did not reveal the nature of such toxicity, which could be mediated by aggregation or non-aggregation. The Western blot analysis indicated that the observed neurodegenerative phenotype in *eraIs1* animals does not have to be necessarily related to αSyn aggregation/fibrillation or membrane-bound αSyn as we detected contradicted insoluble αSyn levels among modifiers of phenotype A and among modifiers of phenotype B. In addition, RNAi of 12 identified modulators also reduced the formation of Q40:YFP inclusions in body wall muscle cells, of which three of these caused the dendrite blebbing in *eraIs1* animals. Thus, we cannot exclude the possibility that some genetic conditions that modulated phenotypes of *eraIs1* animals might have opposite consequences in other model systems. Notably, an increasing number of studies have shown that the formation of αSyn inclusions may be a beneficial mechanism for αSyn neurotoxicity (Wong and Krainc, 2017). We should also take into account that RNAi conditions utilizing the vector L4440 in HT115 bacteria might exert phenotype effects independent of the desired RNAi target and thus reduced YFP foci in some animals exposed to RNAi conditions could be an effect of transgene silencing (De-Souza et al., 2019); some RNAi knockdowns exhibited a decreased fluorescent signal of the soluble Q40:YFP reporter compared to animals exposed to standard OP50 bacteria (Figure 3I). Further investigations of these observed phenotypes and studying the aggregated vs. non-aggregated-related αSyn toxicity in the *eraIs1* model in follow-up studies are needed. Lastly, it should be noted that other tested PD risk genes, which did not modulate *eraIs1* phenotypes in this study, may play a role in αSyn neurotoxicity under different experimental designs or in other model systems or in humans.

Nonetheless, the physiological roles associated with the identified genes also reveal the biological processes that may constitute pathogenetic pathways in sporadic forms of PD. We found that human orthologs of the two identified modulators—*unc-43*/*CAMK2D* and *lfe-2*/*ITPKs*—regulate calcium release from the endoplasmic reticulum into the mitochondria while *C56A3.6*/*MICU3* regulates mitochondrial calcium uptake (Table 1). These data are consistent with a recent observation revealing mammalian *ITPKB* as a protective gene against PD-like phenotypes triggered by mitochondrial calcium uptake (Apicco et al., 2021). We thus conclude that intracellular calcium signaling modulates αSyn neurotoxicity in *C. elegans*. In addition, we identified several specific GTPases and their regulators associated with vesicular trafficking, including synaptic, endosomal, ER/Golgi, and autophagosome/lysosome networks, which altered neuronal αSyn processing. The role of vesicular trafficking in PD is well-documented, and its impairment is one of the leading mechanisms of PD pathogenesis (Hunn et al., 2015). Several genes, such as *sac-2*/*INPP5F* and *nsf-1/NSF* have been shown to modulate PD-like phenotypes in mouse and fruit fly PD models, respectively (Babcock et al., 2015; Cao et al., 2020). Also, another identified modulator *lrk-1*/*LRRK2* and its counterpart *glo-1*/*RAB29* have been previously associated together in PD pathogenesis, but the role of RAB29 in αSyn pathology has not been validated (Kalogeropulou et al., 2020). These findings demonstrate that hypothesis-free identification of candidate PD genes through GWAS can be tracked functionally in a relatively high-throughput way with the *eraIs1* model, to reveal new roles of evolutionary conserved genes in neuronal maintenance upon proteotoxic stress.

Together, reverse genetic screening using a new *C. elegans* PD model system identified 19 functionally interesting PD-risk genes involved in αSyn:Venus toxicity in nematode DA neurons and other nine genes involved in DA neuron maintenance in the αSyn-free system. The obtained data provide a strong foundation for follow-up studies aimed at further characterizing the role of these genes in PD, which represent new potential therapeutic targets for synucleinopathies and other neurodegenerative proteopathies associated with aging.

## Data Availability Statement

The original contributions presented in the study are included in the article/Supplementary Material, further inquiries can be directed to the corresponding author.

## Author Contributions

RV designed, performed, and analyzed the experiments and wrote the manuscript. AH designed and analyzed the experiments. All authors acquired funding and reviewed the manuscript.

## Conflict of Interest

The authors declare that the research was conducted in the absence of any commercial or financial relationships that could be construed as a potential conflict of interest.

## Publisher’s Note

All claims expressed in this article are solely those of the authors and do not necessarily represent those of their affiliated organizations, or those of the publisher, the editors and the reviewers. Any product that may be evaluated in this article, or claim that may be made by its manufacturer, is not guaranteed or endorsed by the publisher.

## Supplementary Material

Supplementary Table 1 List of human Parkinson’s disease (PD) risk genes with respective *Caenorhabditis elegans* orthologs and identified RNA interference (RNAi) phenotype.
